# New Opportunities Beyond Intensive Insulin Therapy for Improving Vascular Health of People Living With Type 1 Diabetes

**DOI:** 10.1002/edm2.70226

**Published:** 2026-04-17

**Authors:** Djordje S. Popovic, Zoltan Kender, Theocharis Koufakis

**Affiliations:** ^1^ Clinic for Endocrinology, Diabetes and Metabolic Disorders, Clinical Centre of Vojvodina, Medical Faculty University of Novi Sad Novi Sad Serbia; ^2^ Department of Endocrinology, Diabetology and Clinical Chemistry (Internal Medicine 1) Heidelberg University Hospital Heidelberg Germany; ^3^ German Center for Diabetes Research (DZD) München‐Neuherberg Germany; ^4^ Second Propaedeutic Department of Internal Medicine, Hippokration General Hospital Aristotle University of Thessaloniki Thessaloniki Greece

**Keywords:** glucagon‐like peptide‐1 receptor agonists, insulin resistance, lifestyle, sodium‐glucose co‐transporter 2 inhibitors (SGLT2i), technology, Type 1 diabetes

Type 1 diabetes (T1D) is an autoimmune disorder characterised by the destruction of pancreatic β‐cells, leading to insulin deficiency, hyperglycaemia and life‐long dependence on exogenous insulin administration. Early intensive glycaemic management is fundamental for reducing and delaying the onset of both microvascular and macrovascular complications of T1D [1, 2]. Despite intensive insulin therapy being the gold standard for glycaemic control in T1D for more than three decades, individuals living with T1D continue to face a significantly higher risk of all‐cause mortality compared with the general population—even when optimal glycaemic targets are achieved, with cardiovascular disease as the leading cause of reduced life expectancy [3]. In this paper, we aim to highlight emerging therapeutic opportunities beyond intensive insulin therapy that may improve vascular outcomes without intention to redefine the pathophysiology of T1D.

One plausible explanation for the persistently elevated risk of cardiovascular mortality among people living with T1D is the rising prevalence of overweight and obesity within this population. For example, an analysis of the U.S. National Health Interview Survey reported that between 2016 and 2021, 34% of adults with T1D were classified as overweight, while 28% were living with obesity [4]. These rates are broadly comparable to those observed in the general population, yet their implications may be particularly important in T1D given the interaction among exogenous insulin therapy, weight gain and cardiometabolic risk. Traditionally, T1D was considered as a condition of lean individuals, yet the prevalence of overweight and obesity in T1D has increased significantly, aligning with the general epidemiological shift, but primarily driven by the widespread use of intensive insulin treatment. Diabetes control and complications trial/epidemiology of diabetes interventions and complications (DCCT/EDIC) study reported that the risk of cardiovascular events among those with the most excessive weight gain in the intensive treatment arm became similar to the one observed in the conventional treatment arm over time [5], reflecting the increased presence of cardiovascular risk factors (visceral adiposity, hypertension, dyslipidemia and insulin resistance [IR]) among individuals with T1D who experienced the greatest weight gain. This was confirmed by the study using the Swedish National Diabetes Registry, which demonstrated that the risk of major cardiovascular disease, heart failure (HF), cardiovascular death and mortality in T1D increases with increasing body mass index (BMI), with associations more apparent in males than in females [6]. Conversely, the study based on the same registry concluded that the presence of BMI > 35 kg/m^2^ among people living with T1D was not independently associated with an increased risk of myocardial infarction, acute coronary events, coronary death and all‐cause mortality compared to those with T1D who had normal weight (BMI: 18.5–25 kg/m^2^). Yet, the presence of underweight (BMI < 18.5 kg/m^2^) was independently associated with an increased risk of coronary and all‐cause mortality compared to the presence of normal weight among individuals living with T1D [7].

Increasing evidence indicates that T1D is a heterogeneous disorder with distinct endotypes that differ in immune mechanisms, genetic background and metabolic profile. These endotypes, ranging from early‐onset immune‐dominant to later‐onset, metabolically mixed forms, have important implications for prediction, prevention and individualised treatment strategies [8, 9]. Recognition of this heterogeneity challenges the traditional view of T1D and supports a more nuanced understanding of its metabolic and cardiovascular consequences. A subset of individuals with T1D develop features of Type 2 diabetes (T2D)—a condition termed ‘double diabetes’. It is characterised by coexisting autoimmune β‐cell failure and pronounced IR, often associated with central obesity, dyslipidemia and hypertension. This phenotype represents a particularly high‐risk group for cardiovascular and renal complications and highlights the convergence of autoimmune and metabolic pathways in T1D pathogenesis [10, 11]. The degree of IR in T1D is heterogeneous and appears to be more pronounced in specific phenotypes, including those with obesity, higher insulin requirements, longer disease duration or other features of metabolic dysfunction. However, since T1D is a condition requiring exogenous insulin administration, quantification of IR is difficult in this setting. The euglycaemic‐hyperinsulinemic clamp is the accepted standard for measurement of insulin sensitivity, but it is not practical for use in everyday clinical practice [12]. On the other hand, the estimated glucose disposal rate (eGDR), based on the routine clinical measures, exhibits good correlation with IR measured by the euglycaemic‐hyperinsulinemic clamp and has been validated for the estimation of insulin sensitivity in T1D [13]. Indeed, there is a strong association between eGDR and all‐cause and cardiovascular mortality in individuals with T1D [14]. Additionally, the presence of metabolic syndrome, the main clinical feature of IR, is associated with the higher prevalence of long‐term complications of diabetes (LTCD) (both microvascular and macrovascular), even in the case of adequate glycaemic control in T1D [15]. Nevertheless, from the pragmatic perspective, the requirement of high insulin doses for attaining adequate glycaemic control, excluding cases in which the insulin administration technique is questionable, hallmarks the presence of IR in T1D. In clinical terms, IR in T1D may be viewed as a relative increase in exogenous insulin requirements to maintain euglycaemia, reflecting reduced tissue insulin sensitivity together with treatment‐related factors specific to this population.

Among people with T1D, excessive visceral fat accumulation is primarily driven by non‐physiological insulin replacement, which leads to peripheral hyperinsulinemia. This is further compounded by the use of insulin preparations whose pharmacokinetic profiles still do not fully replicate physiological basal and prandial insulin needs, as well as by the common practice of defensive snacking to prevent hypoglycaemia [16]. The whole‐body IR emerges in this context, further worsening glycaemic control and promoting proatherogenic dyslipidemia, hypertension, systemic inflammation and vascular injury, thereby contributing to the development of LTCD [17] (Figure 1). The traditional concept of LTCD development in T1D, which focuses on insulin deficiency, chronic hyperglycaemia, treatment‐induced hypoglycaemia and glucose variability, does not fully account for the persistently elevated cardiovascular risk observed in this population. Growing evidence suggests that increased visceral adiposity, the main driver of the IR onset, should be recognised not only as one of the key pathophysiological mechanisms in the progression of LTCD in T1D, but also as an important therapeutic target, similar to T2D, where weight loss represents the most effective treatment approach. From a therapeutic perspective, currently available strategies to improve IR in T1D are largely mediated through weight reduction and improvement in adiposity‐related metabolic dysfunction.

**FIGURE 1:**
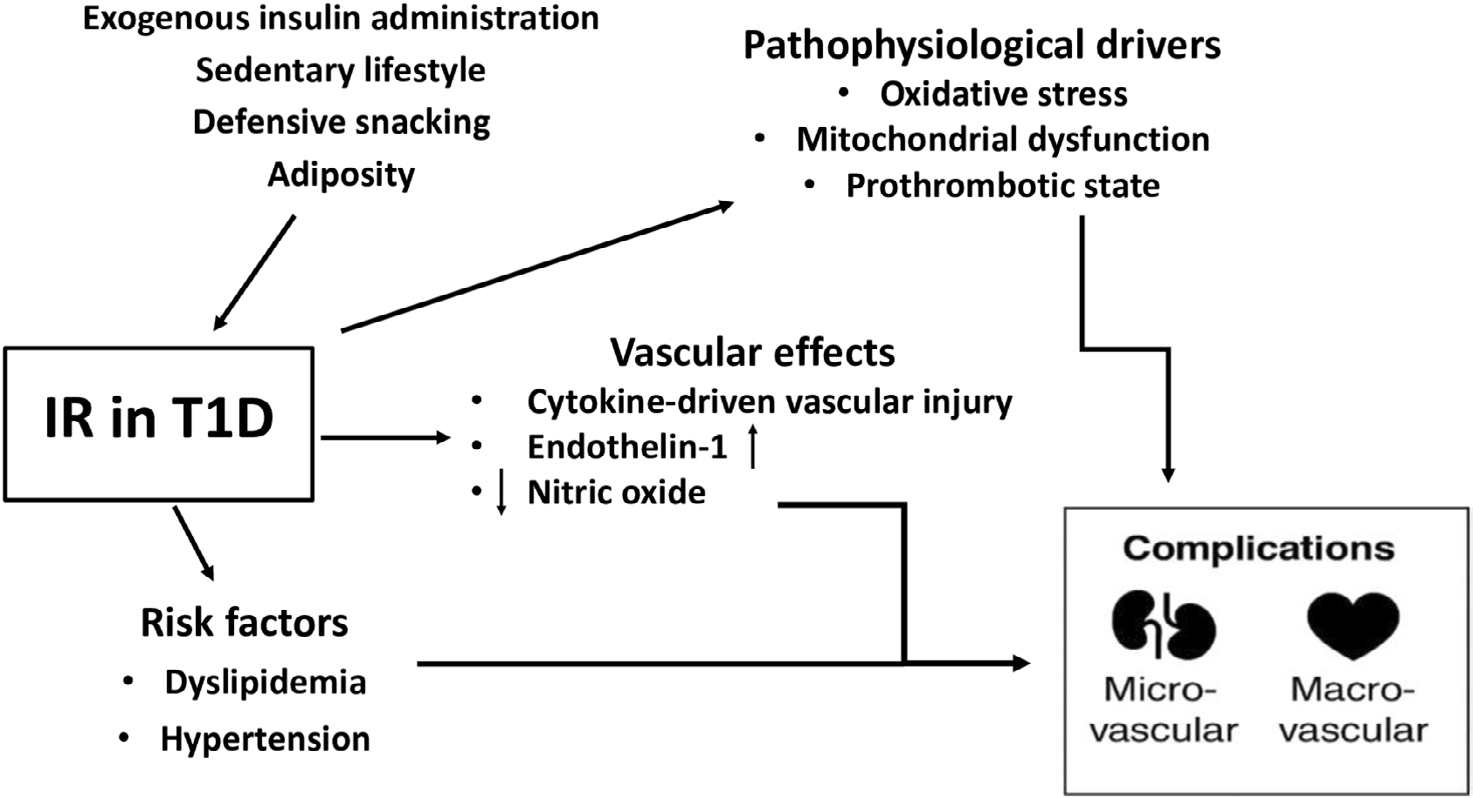
The pathways of insulin resistance‐driven complications in type 1 diabetes. IR, insulin resistance; T1D, Type 1 diabetes.

In addition to insulin therapy, individuals with T1D face substantial lifestyle‐related challenges that complicate disease management and contribute to increased adiposity. Dietary management requires continuous carbohydrate counting and insulin dose adjustment, a process prone to error and associated with both hypo‐ and hyperglycaemia [18]. Defensive snacking to prevent hypoglycaemia is common, often resulting in excess caloric intake and weight gain. Social eating and uncertainty about food composition may further restrict dietary flexibility, occasionally leading to disordered eating behaviours [19]. Physical activity presents another significant barrier. While exercise improves insulin sensitivity and cardiovascular health, it substantially increases the risk of hypoglycaemia during and after activity. The need for frequent glucose monitoring, insulin adjustment and pre‐exercise carbohydrate intake discourages participation in regular or spontaneous exercise [20]. This challenge may also limit weight management efforts. Psychosocial factors exacerbate these difficulties. Diabetes distress, burnout and fear of hypoglycaemia frequently undermine adherence to healthy lifestyle behaviours. Sleep disruption, often due to nocturnal glucose monitoring or fear of nighttime hypoglycaemia, further contributes to impaired metabolic control and reduced quality of life [21]. Finally, individuals with T1D are affected by the same broader societal trends driving obesity in the general population, including sedentary lifestyles and increased consumption of energy‐dense foods [22]. However, the consequences in T1D are magnified due to the delicate balance required among insulin therapy, diet and activity.

Insulin deficiency and chronic hyperglycaemia contribute to vascular damage in T1D through several interrelated mechanisms. Persistent hyperglycaemia promotes the accumulation of advanced glycation end‐products (AGEs), which impair vascular proteins and induce oxidative stress and inflammation [23]. Both hyperglycaemia and AGEs activate protein kinase C, leading to altered blood flow, increased permeability and proinflammatory signalling [24]. Excess glucose flux through the polyol pathway further depletes antioxidant capacity and enhances oxidative stress [25]. Treatment‐induced hypoglycaemia and glycaemic variability also aggravate vascular injury by activating inflammatory, thrombotic and oxidative pathways [26, 27]. Oxidative stress thus represents a central mediator of vascular damage in T1D, promoting endothelial dysfunction, mitochondrial impairment and atherogenesis [28] (Figure 1).

As described above, weight gain in T1D—primarily driven by subcutaneous administration of exogenous insulin and maladaptive eating in response to hypoglycaemia—initiates a vicious cycle of IR [16]. Reduced responsiveness of target tissues to insulin action necessitates progressively higher doses of exogenous insulin, which in turn cause sustained hyperinsulinemia, internalisation and degradation of insulin receptors and a further increase in insulin requirements to achieve comparable glycaemic control [29]. Higher insulin doses not only exacerbate hyperinsulinemia but also heighten the risk of hypoglycaemia, prompting more frequent defensive snacking. Together, these factors perpetuate weight gain, worsen IR and drive the development of dyslipidemia, hypertension and systemic inflammation [17, 29]. At the vascular level, IR stimulates endothelin‐1 production and reduces nitric oxide bioavailability, resulting in vasoconstriction and a prothrombotic state [30]. In parallel, enhanced release of proinflammatory cytokines increases vascular permeability, recruits immune cells to the endothelium and amplifies vascular injury [31] (Figure 1). However, the precise contribution of IR to vascular complications in T1D remains to be fully established, and likely interacts with hyperglycaemia, obesity, insulin exposure and other metabolic disturbances.

Exogenous insulin administration remains the cornerstone of therapy in T1D, as it is essential for survival. However, the isolated management of hyperglycaemia is insufficient to prevent the development of LTCD. Despite the widespread use of next‐generation insulin analogues and increasing accessibility to advanced diabetes technologies, the majority of individuals with T1D are still unable to achieve the recommended glycated haemoglobin (HbA1c) target [32]. Moreover, the risk of all‐cause and cardiovascular mortality remains significantly elevated compared with the general population, even among those achieving optimal glycaemic control [3]. This underscores the need for comprehensive risk factor management, including optimisation of diet, physical activity, antihypertensive therapy and lipid‐lowering agents, as well as pharmacological approaches targeting increased visceral adiposity and IR, which have emerged as one of the key pathophysiological features in the considerable subset of individuals with T1D. Although IR may also be observed in some individuals with T1D in the absence of overt obesity, its clinical relevance is likely greatest in the setting of increased adiposity, which remains the most modifiable driver.

Among the most promising adjunctive therapeutic options in T1D are glucagon‐like peptide‐1 receptor agonists (GLP‐1RA) and sodium‐glucose co‐transporter 2 inhibitors (SGLT2i). These agents have been shown to modestly reduce HbA1c, insulin requirements and body weight in people with T1D [33, 34, 35, 36]. However, these metabolic benefits are counterbalanced by an increased risk of diabetic ketoacidosis (DKA) and, to a lesser extent, hypoglycaemia. Accordingly, GLP‐1RA and SGLT2i currently represent the most relevant adjunctive pharmacological classes under evaluation in this setting. However, neither drug class has received regulatory approval for routine use in T1D. Nevertheless, their established cardiovascular and renal benefits in T2D, together with their potential to address excessive visceral adiposity, IR and metabolic load in T1D, warrant further investigation [37, 38]. Notably, recent randomised controlled trials (RCTs) demonstrated that treatment with semaglutide and tirzepatide, a dual glucose‐dependent insulinotropic polypeptide (GIP) and GLP‐1 receptor agonist, led to modest HbA1c reduction but substantial weight loss and decrease in insulin requirements, without increased risk of DKA and severe hypoglycaemia when compared to placebo, among individuals living with T1D and obesity [39, 40]. These findings further support a weight‐centred therapeutic paradigm in T1D and provide a rationale for continued evaluation of newer incretin‐based therapies, including dual agonists. On the other hand, a robust retrospective cohort study reported that use of adjunctive therapy with SGLT2i was associated with lower risk of HF, chronic kidney disease (CKD) and hospitalisation for any cause but higher risk of DKA and urinary tract infection/pyelonephritis compared with adjunctive use of GLP‐1RA among individuals living with T1D after the 5‐year follow‐up [41]. However, future studies focused on refining patient selection, optimising dosing and exploring combination strategies to improve safety and maximise cardiometabolic benefits are still needed.

Alongside pharmacological advances, lifestyle modification remains a cornerstone for improving weight management, IR and cardiorenal outcomes in T1D. Dietary strategies such as reducing intake of ultra‐processed and energy‐dense foods, increasing dietary fibre and adopting a Mediterranean‐style diet have been shown to improve insulin sensitivity and support weight management [42]. Regular physical activity is equally important, as both aerobic and resistance training enhance peripheral glucose uptake and reduce IR by increasing skeletal muscle insulin sensitivity. Moreover, structured exercise interventions have demonstrated benefits in reducing cardiovascular risk factors, improving lipid profiles and lowering blood pressure in people with T1D [43].

Combining individualised nutrition counselling with supervised or technology‐assisted exercise programs may further enhance adherence and mitigate barriers such as fear of hypoglycaemia, thereby making lifestyle interventions more sustainable [44].

These non‐pharmacological strategies, when integrated with adjunctive pharmacotherapy, represent a synergistic approach to mitigating excessive visceral adiposity and consequent IR (Figure 2), thus reducing long‐term cardiovascular and renal risk in T1D.

**FIGURE 2:**
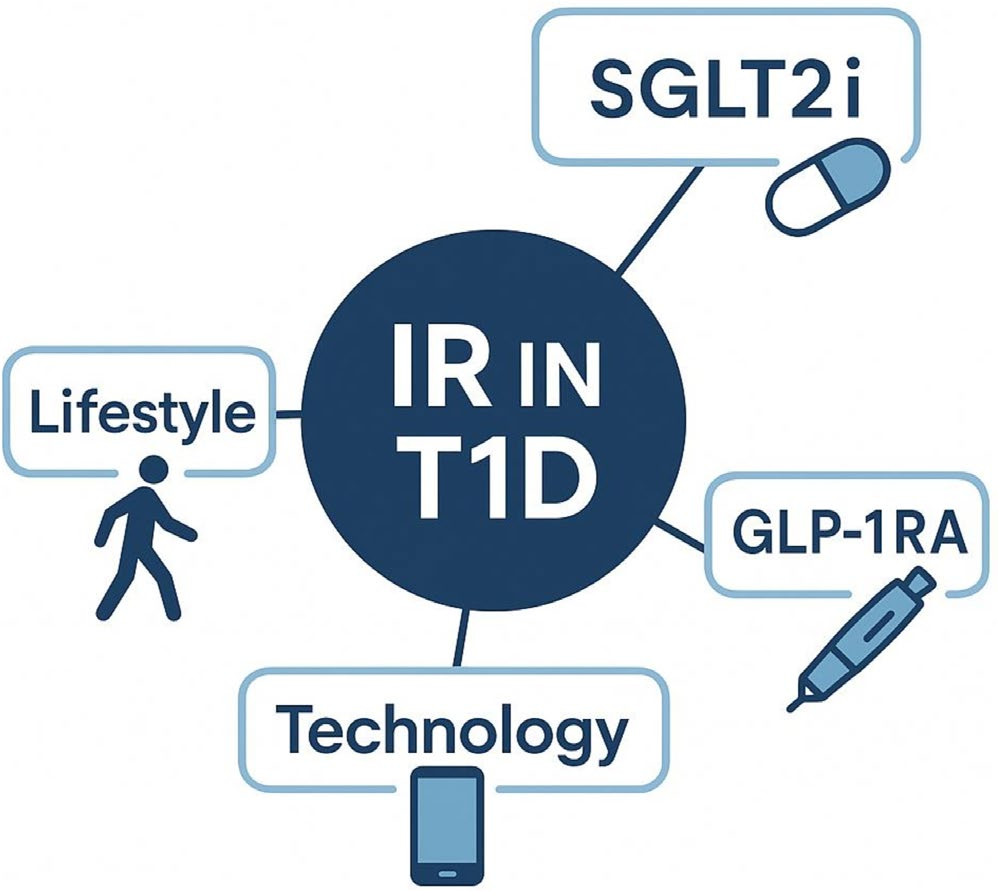
Methods to alleviate insulin resistance in people living with type 1 diabetes. IR, insulin resistance; T1D, Type 1 diabetes; SGLT2i, sodium‐glucose cotransporter 2 inhibitors; GLP‐1RA, glucagon‐like peptide 1 receptor agonists.

The persistence of shortened life expectancy in T1D—primarily driven by elevated cardiovascular mortality—underscores the need to move beyond a strictly ‘glucocentric approach’. While optimal glycaemic control remains essential, it is not sufficient on its own to fully mitigate the risk of LTCD. Novel therapeutic strategies are therefore required, including the adjunctive use of selected non‐insulin antidiabetic agents, particularly GLP‐1RA and SGLT2i, owing both to their glycaemic and non‐glycaemic effects. Namely, although originally introduced as glucose‐lowering agents, both GLP‐1RA and SGLT2i also exert broader cardiometabolic effects beyond glycaemic control. Importantly, selection of the most appropriate adjunctive treatment should be based on the specific phenotypes of T1D, considering available data from RCTs conducted among persons living with T1D, and firmly established effects of these drugs in the settings of T2D, but also among individuals without diabetes. Individuals more eligible for treatment with GLP‐1RA would be those living with obesity, individuals with residual endogenous insulin secretion, or the ones with an established atherosclerotic cardiovascular disease (ASCVD)/at high risk for future ASCVD development, with the necessary caution, especially regarding the potential risk of severe hypoglycaemia [37]. On the other hand, individuals with or at high risk of, CKD and/or HF would benefit more from the adjunctive therapy with SGLT2i, with due diligence in terms of individual risk for SGLT2i‐related DKA development [38]. These adjunctive approaches should preferably be implemented in specialist settings with careful patient selection, education and monitoring and in parallel with strict management of other modifiable risk factors such as hypertension, dyslipidemia, obesity and sedentary behaviour.

Beyond pharmacotherapy, comprehensive care should also incorporate innovative approaches to support lifestyle modification and patient self‐management. The integration of artificial intelligence–driven decision‐support tools, smartphone applications, continuous glucose monitoring platforms and digital coaching programs can facilitate personalised feedback, improve adherence to diet and physical activity recommendations and reduce the burden of daily self‐management. Coupled with structured education and multidisciplinary care, these digital solutions have the potential to transform T1D management by promoting sustainable behavioural change and improving long‐term outcomes.

In summary, addressing the excess cardiovascular risk in T1D requires a multifaceted strategy that combines advanced pharmacological therapies with digital health innovations and lifestyle interventions, thereby shifting towards a more holistic and patient‐centred model of care of T1D. Adjunctive therapies should currently be considered mainly in selected individuals with T1D, particularly those with obesity, high insulin requirements or increased cardiovascular or renal risk and ideally within specialist care settings. Future trials should further define the phenotypes most likely to benefit and assess long‐term vascular outcomes.

## Author Contributions

**Djordje S. Popovic:** conceptualization, writing – original draft, writing – review and editing. **Zoltan Kender:** writing – original draft, writing – review and editing. **Theocharis Koufakis:** writing – original draft, writing – review and editing.

## Funding

The authors have nothing to report.

## Conflicts of Interest

Djordje S. Popovic declares associations to Abbott, ADOC Pharma, Alkaloid, AstraZeneca, Bayer, Boehringer‐Ingelheim, Berlin‐Chemie, Eli Lilly, Galenika, Krka, Merck, Novo Nordisk, PharmaSwiss, Sanofi‐Aventis, Servier, Viatris and Wörwag Pharma. Zoltan Kender received grants from the German Center of Diabetes Research (DZD) and from the German Society of Diabetes (DDG). Theocharis Koufakis has received honoraria for lectures from AstraZeneca, Boehringer Ingelheim, Pharmaserve Lilly and Novo Nordisk, for advisory boards from Novo Nordisk, and has participated in sponsored studies by Eli Lilly and Novo Nordisk. No conflicts of interest reported by any author herein is directly related to the work presented herein.

## Data Availability

Data sharing not applicable to this article as no datasets were generated or analysed during the current study.
